# Supplementary material for: Identifying host regulators and inhibitors of liver stage malaria infection using kinase activity profiles
Source: Nat Commun. 2017 Nov 1;8:1232. doi: 10.1038/s41467-017-01345-2 (PMC5663700; doi:10.1038/s41467-017-01345-2)
Supplement: Supplementary file 1 — Supplementary Information [file 41467_2017_1345_MOESM1_ESM.pdf]

**SUPPLEMENTARY INFORMATION**

**Supplementary Figure 1**

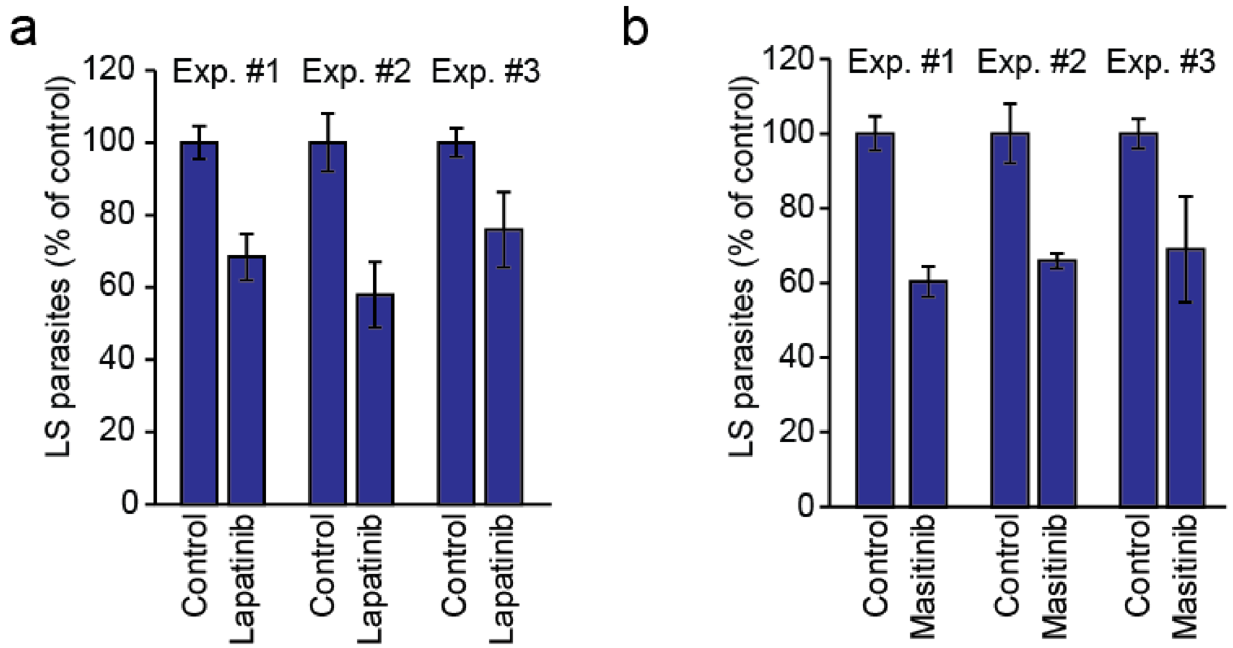


**Parasite clearance by kinase inhibitors is consistent over multiple biological replicates.** 150,000 Hepa 1-6 cells were infected with 50,000 *P. yoelii* parasites, and then treated with (a) Lapatinib or (b) Masitinib at 500nm 1.5hpi. Parasite burden was evaluated by microscopy by staining of *P. yoelii* HSP70 and DAPI for nuclear visualization at 24hpi. Data shown is the average of 3-5 independent experiments. Error bars represent standard deviation of independent experiments.

**Supplementary Figure 2**

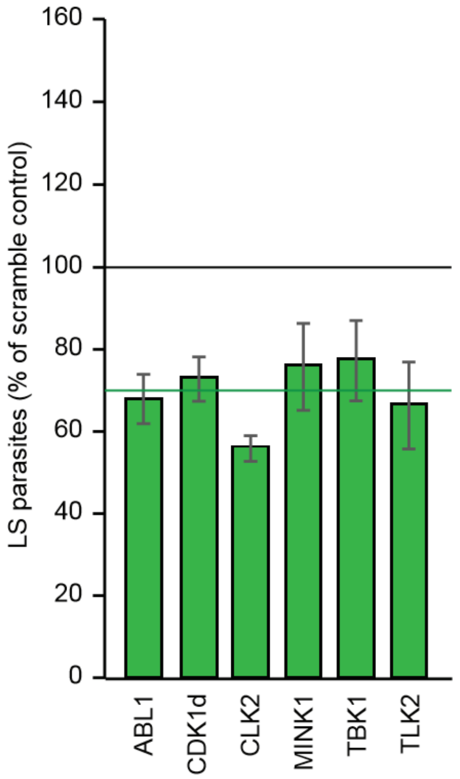


**LS burden after knockdown of negative hit kinases:** Bar graph depicting LS development in cells with shRNA-mediated knockdown of a subset of kinases not predicted to play a role in LS infection. Values are normalized to non-treated parasites which are indicated by solid black line. Threshold for validation is ≤70% remaining LS burden after kinase knockdown, indicated by blue line. Data is representative of 3 independent experiments. Error bars represent standard deviation of analytical replicates.

**Supplementary Figure 3**

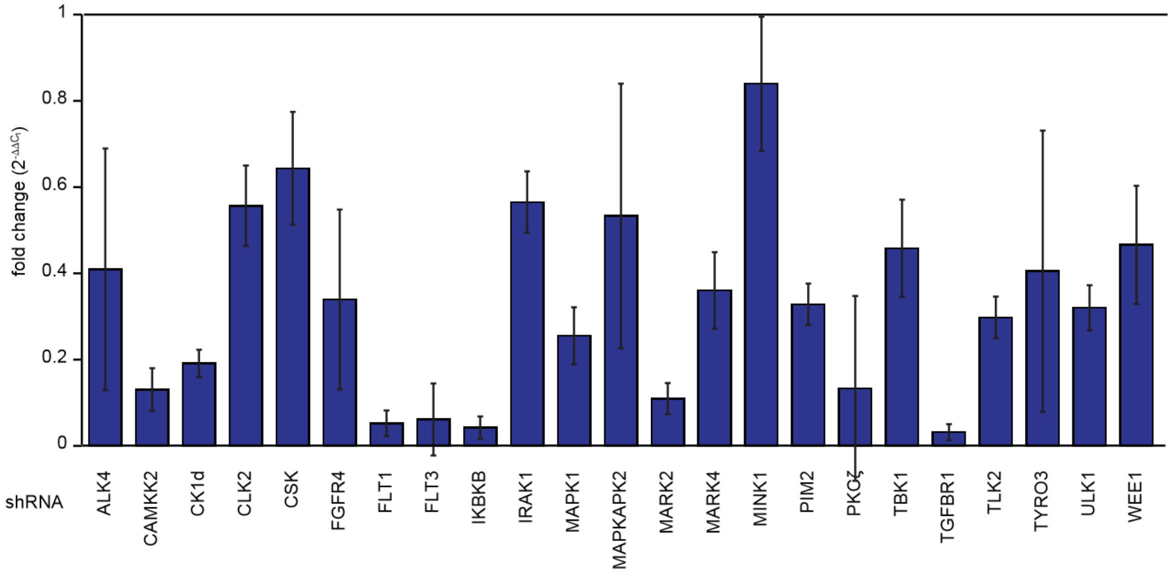


**Selective knockdown of hit kinases can be achieved using lentivirus-mediated shRNA.** Bar graph depicting relative gene knockdown compared to non-targeting control shRNA. Hepa 1-6 cells were transduced with lentivirus expressing shRNA constructs selectively targeting hit kinases, or a non-targeting control. Mean knockdown level was determined using qPCR. Values are normalized to non-targeting control which is indicated by solid black line. Data is representative of 3 independent experiments. Error bars represent propagated standard deviation of analytical replicates.

**Supplementary Figure 4**

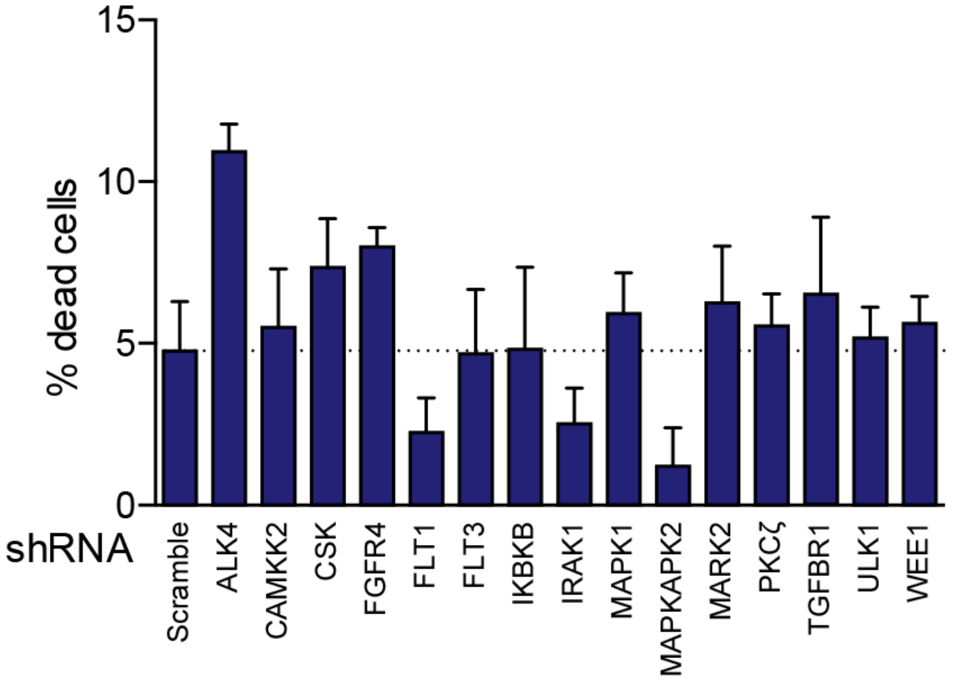


**Selective knockdown of hit kinases by lentivirus-mediated shRNA does not induce substantial cell death.** Bar graph depicting percentage of trypan-blue positive cells after shRNA knockdown compared to non-targeting control shRNA (scramble). Hepa 1-6 cells were transduced with lentivirus expressing shRNA constructs selectively targeting hit kinases, or a non-targeting control. Percentage of dead cells was assessed by trypan blue staining. Data is representative of two independent experiments. Error bars represent standard deviation of three technical replicates.

**Supplementary Table 1**

| | Kinase Inhibitor | CAS # |
|---|---|---|
| Inhibitors used to train algorithm | Aminopurvanolol A | 220792-57-4 |
| | Bosutinib | 380843-75-4 |
| | Casein kinase I inhibitor D44 | 301836-43-1 |
| | CDK2 inhibitor IV | 444723-13-1 |
| | CDK4 inhibitor | 546102-60-7 |
| | Dasatinib | 302962-49-8 |
| | EGFR/ErbB2/ErbB4 inhibitor | 881001-19-0 |
| | Go 6976 | 136194-77-9 |
| | Go 6983 | 133053-19-7 |
| | GSK inhibitor IX | 667463-62-9 |
| | GSK-3 Inhibitor X | 740841-15-0 |
| | GSK-3 Inhibitor XIII | 404828-08-6 |
| | H89 | 127243-85-0 |
| | Imatinib | 220127-57-1 |
| | JNK inhibitor II | 129-56-6 |
| | K252a | 97161-97-2 |
| | Lapatinib | 231277-92-2 |
| | Lck inhibitor | 213743-31-8 |
| | Masitinib | 790299-79-5 |
| | PKR inhibitor | 608512-97-6 |
| | ROCK inhibitor (Y-27632) | 146986-50-7 |
| | SB218078 | 135897-06-2 |
| | Sorafenib | 284461-73-0 |
| | Staurosporine | 62996-74-1 |
| | SU11274 | 658084-23-2 |
| | Tofacitnib | 477600-75-2 |
| | TWS119 | 601514-19-6 |
| | Vandetanib | 443913-73-3 |
| Inhibitors omitted from further analysis | JAK inhibitor I | 457081-03-7 |
| | Erlotinib | 183319-69-9 |
| | AMPK Inhibitor; Compound C | 866405-64-3 |
| | Staurosporine n benzoyl | 120685-11-2 |
| | GSK-3b inhibitor I | 327036-89-5 |
| | SU6656 | 330161-87-0 |
| | Dovitinib | 405169-16-6 |
| | Gefitinib | 184475-35-2 |
| | Nilotinib | 641571-10-0 |

| | | |
|---|---|---|
| Inhibitors chosen for follow up analysis | Cdk2 inhibitor IV | 444723-13-1 |
| | Cdk1/2 inhibitor III | 443798-55-8 |
| | SB505124 | 356559-13-2 |
| | LY364947 | 396129-53-6 |
| | VX-680 | 639089-54-6 |
| | Roscovitine | 186692-46-6 |
| | Sunitinib | 557795-19-4 |

**Panel of kinase inhibitors tested against *Plasmodium***. Kinase inhibitors used to train algorithm, those omitted from analysis, and those chosen for follow up analysis. Drug names and CAS numbers are included.

**Supplementary Table 2**

| Name | Exp. 1 | Exp. 2 | Exp. 3 | Exp. 4 | Mean LS burden (% of DMSO control) | Standard Deviation |
|---|---|---|---|---|---|---|
| Aminopurvanolol A | 57.00 | 74.76 | 67.96 | | 66.57 | 8.96 |
| Bosutinib | 75.11 | 72.33 | 92.05 | | 79.83 | 10.67 |
| Casein kinase I inhibitor D44 | 135.86 | 107.69 | 115.53 | 105.28 | 116.09 | 13.89 |
| CDK2 inhibitor IV; NU6140 | 128.00 | 101.94 | 89.65 | | 106.53 | 19.58 |
| CDK4 inhibitor | 35.45 | 33.50 | 57.14 | | 42.03 | 13.13 |
| Dasatinib | 51.90 | 88.83 | 79.92 | | 73.55 | 19.27 |
| EGFR/ErbB2/ErbB4 inhibitor | 120.00 | 116.02 | 96.07 | | 110.70 | 12.82 |
| Go 6976 | 59.92 | 91.26 | 79.17 | | 76.78 | 15.81 |
| Go 6983 | 60.34 | 52.43 | 73.86 | | 62.21 | 10.84 |
| GSK inhibitor IX (BIO) | 37.13 | 56.21 | 76.70 | 76.09 | 61.53 | 18.85 |
| GSK-3 Inhibitor X | 33.21 | 47.57 | 76.40 | 39.81 | 49.25 | 19.03 |
| GSK-3 Inhibitor XIII | 72.00 | 31.11 | 73.86 | 60.19 | 59.29 | 19.74 |
| H89 | 36.57 | 60.23 | 78.78 | 64.08 | 59.91 | 17.50 |
| Imatinib (mesylate) | 41.77 | 45.27 | 81.63 | 65.05 | 58.43 | 18.56 |
| JNK inhibitor II (SP600125) | 61.18 | 38.26 | 37.86 | | 45.77 | 13.35 |
| K252a | 0.00 | 14.00 | 22.15 | | 12.05 | 11.20 |
| Lapatinib | 68.35 | 57.95 | 75.92 | | 67.41 | 9.02 |
| Lck inhibitor | 65.00 | 42.43 | 54.69 | | 54.04 | 11.30 |
| Masitinib | 60.34 | 65.91 | 68.98 | | 65.08 | 4.38 |
| PKR inhibitor | 30.10 | 68.89 | 57.55 | | 52.47 | 19.46 |
| ROCK inhibitor (Y-27632) | 77.64 | 55.30 | 81.16 | | 71.37 | 14.02 |
| SB218078 | 41.42 | 80.03 | 67.76 | | 63.07 | 19.73 |
| Sorafenib | 51.48 | 64.02 | 40.41 | 79.30 | 58.80 | 16.72 |
| Staurosporine | 0.00 | 0.00 | 0.00 | | 0.00 | 0.00 |
| SU11274 | 30.30 | 40.82 | 9.33 | | 26.82 | 16.03 |
| Tofacitnib | 75.95 | 49.62 | 47.35 | | 57.64 | 15.90 |
| TWS119 | 63.29 | 34.85 | 50.20 | | 49.45 | 14.24 |
| Vandetanib | 75.11 | 37.88 | 52.24 | | 55.08 | 18.77 |

**Mean LS burden in response to kinase inhibitors:** Data from each experimental replicate assessing LS burden in response to kinase inhibitors used for training dataset. Three individual experiments were performed testing the efficacy of each compound.

An additional independent experiment was performed if the standard deviation was ≥ 20% of the sample mean for the initial three replicates.

**Supplementary Table 3**

| Gene Symbol | Gene Description | NCBI Gene ID | Sigma MISSION shRNA clone ID |
|---|---|---|---|
| ABL1 | c-abl oncogene 1, receptor tyrosine kinase | 11350 | TRCN0000321753 |
| | | | TRCN0000023356 |
| ALK4 | activin A receptor, type 1B | 11479 | TRCN0000345030 |
| | | | TRCN0000345027 |
| | | | TRCN0000022574 |
| | | | TRCN0000022575 |
| CAMKK2 | calcium/calmodulin-dependent protein kinase kinase 2, beta | 207565 | TRCN0000028815 |
| | | | TRCN0000276649 |
| CK1d | casein kinase 1, delta | 104318 | TRCN0000361907 |
| | | | TRCN0000023770 |
| CLK2 | CDC-like kinase 2 | 12748 | TRCN0000023057 |
| | | | TRCN0000023058 |
| CSK | c-src tyrosine kinase | 12988 | TRCN0000023736 |
| | | | TRCN0000321790 |
| FGFR4 | fibroblast growth factor receptor 4 | 14186 | TRCN0000023564 |
| | | | TRCN0000023567 |
| FLT1 | FMS-like tyrosine kinase 1 | 14254 | TRCN0000009606 |
| | | | TRCN0000009610 |
| | | | TRCN0000009609 |
| FLT3 | FMS-like tyrosine kinase 3 | 14255 | TRCN0000023742 |
| | | | TRCN0000023743 |
| IKBKB | inhibitor of kappaB kinase beta | 16150 | TRCN0000026913 |
| | | | TRCN0000026894 |
| IRAK1 | interleukin-1 receptor-associated kinase 1 | 16179 | TRCN0000271170 |
| | | | TRCN0000284313 |
| | | | TRCN0000023666 |
| MAPK1 | mitogen activated protein kinase 1 | 26413 | TRCN0000023160 |
| | | | TRCN0000054730 |
| MAPKAPK2 | MAP kinase-activated protein kinase 2 | 17164 | TRCN0000232383 |
| | | | TRCN0000232382 |
| MARK2 | MAP/microtubule affinity-regulating kinase 2 | 13728 | TRCN0000023985 |
| | | | TRCN0000023987 |
| | | | TRCN0000321808 |

| | | | |
|---|---|---|---|
| MARK4 | MAP/microtubule affinity-regulating kinase 4 | 232944 | TRCN0000374403 |
| | | | TRCN0000374404 |
| MINK1 | misshapen-like kinase 1 (zebrafish) | | TRCN0000025335 |
| | | | TRCN0000025338 |
| PIM2 | proviral integration site 2 | 18715 | TRCN0000024158 |
| | | | TRCN0000024156 |
| PRKCZ | protein kinase C, zeta | 18762 | TRCN0000274628 |
| | | | TRCN0000022873 |
| TBK1 | TANK-binding kinase 1 | 56480 | TRCN0000323444 |
| | | | TRCN0000345204 |
| TGFRR1 | transforming growth factor, beta receptor I | 21812 | TRCN0000322044 |
| | | | TRCN0000322046 |
| TLK2 | tousled-like kinase 2 (Arabidopsis) | 24086 | TRCN0000322067 |
| | | | TRCN0000026998 |
| TYRO3 | TYRO3 protein tyrosine kinase 3 | 22174 | TRCN0000361563 |
| | | | TRCN0000361494 |
| | | | TRCN0000361566 |
| | | | TRCN0000361564 |
| ULK1 | Unc-51 like kinase 1 (C. elegans) | 22241 | TRCN0000361568 |
| | | | TRCN0000028768 |
| WEE1 | wee 1 homolog (S. pombe) | 22390 | TRCN0000025673 |
| | | | TRCN0000025670 |

**shRNA constructs used to knock down host kinases**. Table of shRNA constructs used to generate lentivirus and induce knockdown of select host kinases. Gene name, gene IDs, and MISSION clone numbers are included.

**Supplementary Table 4:**

| Gene Name | Forward Primer (5' to 3') | Reverse Primer (5' to 3') |
|---|---|---|
| Abl1 | AGCCGCTTCAACACTCTGG | ACACCGTAGATAGTGGGCTTG |
| Alk4 | TTCTTCCCCCTTGTTGTCCTC | ACAGGTGTAGTTGGTCTGTAGG |
| Camkk2 | TCATGTGTCTCTAGCCAGCC | TGACCACGATGAAGGATTCCAT |
| Ck1d | ACGCCGGGATCGAGAAGAA | CCGACCGGGAATCTGTGAG |
| Clk2 | CGAACACTATCAGAGCCGAAAG | GAACGTGGTAGCTGTCCTCC |
| Csk | TTCCCTTCTGCAAAGGAGATGT | ACCAGGGCATAAGGCTGAGT |
| Fgfr4 | GCTCGGAGGTAGAGGTCTTGT | CCACGCTGACTGGTAGGAA |
| Flt1 | TGGATGAGCAGTGTGAACGGCT | GCCAAATGCAGAGGCTTGAACG |
| Flt3 | GCTGTACGTGCTAAGAAGACC | AGCATCTGATGTCTGTTCCGA |
| Ikbkb | ACAGCCAGGAGATGGTACG | CAGGGTGACTGAGTCGAGAC |
| Irak1 | CCACCCTGGGTTATGTGCC | GAGGATGTGAACGAGGTCAGC |
| Mapk1 | CAGGTGTTCGACGTAGGGC | TCTGGTGCTCAAAAGGACTGA |
| Mapkapk2 | TTCCCCCAGTTCCACGTCA | GCAGCACCTTCCCGTTGAT |
| Mark2 | CTACCCACGCTGAACGAAAGG | GTAGTTGCCAATATGGGGCTG |
| Mark4 | TGGGCAGTGGACGATCTTC | CTTCCCGATGGTCCTTAGCAG |
| Mink1 | CCACCTACTATGGGGCCTTTA | AGCACCGCAGAACTCCATC |
| Pim2 | TTCAGCGGGCTCAATATACGC | CCAAGTCGGTATTCGGCCTC |
| Prkcz | GCGTGGATGCCATGACAAC | AATGATGAGCACTTCGTCCCT |
| Tbk1 | ACTGGTGATCTCTATGCTGTCA | TTCTGGAAGTCCATACGCATTG |
| Tgfbr1 | TCTGCATTGCACTTATGCTGA | AAAGGGCGATCTAGTGATGGA |
| Tlk2 | AGCCCTGGCAGAAGTGTTC | GCGGTAAGGGATTGGATAAGGAA |
| Tyro3 | GCCTCCAAATTGCCCGTCA | CCAGCACTGGTACATGAGATCA |
| Ulk1 | AAGTTCGAGTTCTCTCGCAAG | CGATGTTTTCGTGCTTTAGTTCC |
| Wee1 | GTCGCCCGTCAAATCACCTT | GAGCCGGAATCAATAACTCGC |
| Gapdh | AGGTCGGTGTGAACGGATTT | GGGGTCGTTGATGGCAACA |

**qPCR primers used to assess knockdown of hit host kinases**. Level of shRNA mediated knockdown of host kinases was assessed by qPCR. Gene names, gene IDs, and primer sequences are included.

**Supplementary Note 1**

Below is the script used to perform the regression analysis included in the manuscript.

The code can be run in MATLAB™.

```
Ts=importdata('data.csv');

Ts.colheaders = Ts.textdata(1,3:end);

D2T = importdata('drugs_data.csv');

D2T.kinaseIDs = D2T.textdata(1,2:end);

D2T.drugsIDs = D2T.textdata(2:end,1);

D2T = rmfield(D2T,{'textdata'});

minLambda = 0.05;

maxLambda = 50;

nLam = 100;

lambdas = minLambda*nthroot(maxLambda/minLambda,nLam - 1).^((1:nLam) -
1);


alphaVals = 0.1:0.1:1;

coeffs = zeros(size(Ts.data,2) - 1, size(alphaVals,2));

preds = zeros(size(D2T.data,1), size(alphaVals,2));

MSEs = zeros(nLam,size(alphaVals,2));

constants = zeros(size(alphaVals,2),1);

i = 1;

for alpha = alphaVals

    [B, FitInfo] = lasso(Ts.data(:,2:end),Ts.data(:,1),'CV',
'resubstitution' ,'Alpha', alpha,'MCReps',1, 'Lambda', lambdas,
'PredictorNames', Ts.textdata(1,3:end));

    MSEs(1:size(FitInfo.MSE,2),i) = FitInfo.MSE';

    [~, indx]  = min(FitInfo.MSE);

    coeffs(:,i) = B(:, indx);
```

```
    B0 = B(:,indx);

    cnst = FitInfo.Intercept(indx);

    constants(i) = cnst;

    B1 = [cnst; B0];

    preds(:,i) = glmval(B1, D2T.data,'identity');

    i = i+1;

end

alphaLevels = cellstr(strcat({'alpha '},string(alphaVals)));

alphaLevels = regexprep(alphaLevels,'[^a-zA-Z0-9]','_');

kinNames = regexprep(Ts.textdata(1,3:end),'[^a-zA-Z0-9]','_');

alphaCoeffs = array2table(coeffs, 'RowNames', kinNames,
'VariableNames', alphaLevels);

inhNames = regexprep(D2T.drugsIDs, '[^a-zA-Z0-9]', '_');

inhPreds = array2table(preds, 'RowNames', inhNames, 'VariableNames',
alphaLevels);

inhPreds.Mean = mean(inhPreds{:,:},2);

inhPreds = sortrows(inhPreds, 'Mean');

MSEVals = array2table(MSEs, 'VariableNames', alphaLevels);

MSEVals.Lambda = lambdas';

MSEVals = [MSEVals(:,size(alphaVals,2)+1)
MSEVals(:,1:size(alphaVals,2))];


toc


writetable(inhPreds, 'Inhibitor_Predictions.csv', 'WriteRowNames',
true)

writetable(alphaCoeffs, 'Kinase_Predictions.csv', 'WriteRowNames',
true)

%writetable(MSEVals, 'MSE.csv')
```
