# Supplementary material for: Identifying host regulators and inhibitors of liver stage malaria infection using kinase activity profiles
Source: Nat Commun. 2017 Nov 1;8:1232. doi: 10.1038/s41467-017-01345-2 (PMC5663700; doi:10.1038/s41467-017-01345-2)
Supplement: Supplementary file 3 — Description of Additional Supplementary Files [file 41467_2017_1345_MOESM3_ESM.pdf]

## Description of Additional Supplementary Files

File Name: Supplementary Data 1
Description: **Kinase predictions at various values of alpha:** All kinases assessed using multivariate regression and their predicted coefficients at various alpha values between 0.1 and 1.0. Kinases described in the paper were chosen at alpha=0.8

File Name: Supplementary Data 1
Description: **Predicted efficacies of untested kinase inhibitors:** Elastic net predictions of residual LS burden in response to complete set of kinase inhibitors with publically available activity profiles. Predicted LS burden is reported as percent of non- (206) 256-7200 cidresearch.org 307 Westlake Ave North, Suite 500 Seattle, WA 98109 treated control. Kinase inhibitors are sorted from highest to lowest predicted efficacy against LS Plasmodium yoelii infection.
